# Network Meta-Analysis of Acupoint Catgut Embedding in Treatment of Simple Obesity

**DOI:** 10.1155/2022/6408073

**Published:** 2022-05-23

**Authors:** Zhuo-yuan Wang, Xiao-yan Li, Xiao-jun Gou, Chun-lan Chen, Zun-yuan Li, Chuang Zhao, Wen-ge Huo, Yu-hong Guo, Yan Yang, Zhi-dan Liu

**Affiliations:** ^1^Baoshan Hospital affiliated to Shanghai University of Traditional Chinese Medicine, Shanghai 201999, China; ^2^Baoshan District Hospital of Integrated Traditional Chinese and Western Medicine of Shanghai, Shanghai 201999, China

## Abstract

**Objective:**

To evaluate the clinical efficacy of acupoint catgut embedding in the treatment of simple obesity through network meta-analysis.

**Methods:**

PubMed, Cochrane, Embase, China National Knowledge Infrastructure (CNKI), Wanfang, and VIP database (VIP) were searched by using computer from 2011 to August 2021, and 35 RCT studies were retrieved. The quality of the literature was evaluated using the modified Jadad scoring table, and Stata 15.0 software was used for traditional meta-analysis and network meta-analysis.

**Results:**

Thirty-five RCTs (3040 cases in total) were included. Acupoint embedding, acupuncture, electroacupuncture, TCM, acupoint embedding + acupuncture, acupoint embedding + exercise diet therapy, acupoint embedding + TCM, exercise diet therapy, acupoint embedding + moxibustion, and acupoint embedding + cupping were investigated in the studies. The results of network meta-analysis were as follows: in terms of total effective rate, acupoint catgut embedding was superior to acupuncture, electroacupuncture, and exercise diet therapy (*P* < 0.05); electroacupuncture, acupoint catgut embedding + acupuncture, acupoint catgut embedding + exercise diet therapy, acupoint catgut + TCM, acupoint catgut + moxibustion, and acupoint catgut + cupping were superior to acupuncture (*P* < 0.05); acupoint catgut + moxibustion was superior to electroacupuncture (*P* < 0.05); acupoint catgut + TCM, acupoint catgut + moxibustion, and acupoint catgut + cupping were superior to TCM treatment (*P* < 0.05); and electroacupuncture, acupoint catgut, acupoint catgut + acupuncture, acupoint catgut + exercise diet therapy, acupoint catgut + TCM, acupoint catgut embedding + moxibustion, and acupoint catgut embedding + cupping were superior to sports diet therapy (*P* < 0.05). Regarding weight loss, acupuncture treatment was superior to acupoint catgut embedding therapy (*P* < 0.05); acupoint catgut embedding + exercise diet therapy, acupoint catgut embedding + TCM, acupoint catgut embedding + moxibustion, and acupoint catgut embedding + cupping were superior to acupuncture and electroacupuncture treatment (*P* < 0.05); acupoint catgut embedding + exercise diet therapy, acupoint catgut embedding + TCM, and acupoint catgut embedding + moxibustion were superior to TCM treatment (*P* < 0.05); and acupoint catgut embedding, acupoint catgut embedding + acupuncture, catgut embedding + exercise diet therapy, acupoint catgut embedding + TCM, acupoint catgut embedding + moxibustion, and acupoint catgut embedding + cupping were superior to exercise diet therapy (*P* < 0.05). In terms of BMI reduction, acupoint catgut embedding + moxibustion and acupoint catgut embedding + cupping were more evident than acupuncture treatment (*P* < 0.05); and acupoint catgut embedding + moxibustion was more evident than electroacupuncture treatment (*P* < 0.05).

**Conclusion:**

Acupoint catgut embedding and its combination with other therapies are the first choice for the treatment of simple obesity.

## 1. Introduction

Obesity is a chronic disease due to excessive accumulation or abnormal distribution of fat in the body [1]. Simple obesity is a kind of obesity caused by excessively more intake than consumption, excluding other diseases or medical factors [2]. Simple obesity is now an epidemic health problem, leading to higher incidence of other diseases. Excessive fat accumulation in the body is an important manifestation of obese people, resulting in a higher body mass index (BMI) than normal [3]. Over the past few decades, obesity has been considered to be the result of unbalanced intake and consumption of high-calorie diet. The survey shows that from 1993 to 2015, the prevalence of overweight and obesity, especially abdominal obesity, increased significantly among Chinese adults [4]. Obesity is the main risk factor of dyslipidemia and cardiovascular disease (CVD) [5]. At present, there are many treatment methods for obesity. Some literature studies show that acupoint catgut embedding and joint use with other therapies can improve the effective rate of treatment for simple obesity and reduce the BMI of patients [6]. This study used network meta-analysis to compare the efficacy of acupoint catgut embedding and its combination with other methods in the treatment of simple obesity, so as to provide some evidence support for clinical adjuvant therapy.

## 2. Data and Methods

### 2.1. Methods

PubMed, Cochrane, Embase, China National Knowledge Infrastructure (CNKI), Wanfang, and VIP Database (VIP) were selected as databases. “Catgut embedding at acupoints”, “simple obesity”, and “obesity” were Chinese and English search terms. The combination of subject headings and free words was the retrieval method. Title, abstract and keywords or Title, Abstract and Keywords were used as search entries. From the establishment of the database to August 2021 was the time for publication of the literature.

### 2.2. Inclusion and Exclusion Criteria

The inclusion criteria of the literature were as follows: (1) patients who met the diagnostic criteria established by the Fifth National Obesity Academic Research Conference [7]; (2) type of study: RCTs were included according to the criteria in the Cochrane Collaborative Workbook; (3) intervention measures: the baseline data were complete, and at least two groups were included. Catgut embedding therapy was used in the test group, and the main indicators were drug response rate, body weight change, and BMI; and (4) languages were limited to Chinese and English.

The exclusion criteria were as follows: (1) nonrandomized control; (2) literature with a sample size of less than 10 or repeated publications; (3) studies with incomplete data and lack of rigorous study design; and (4) studies with unclear efficacy outcome evaluation and analysis.

### 2.3. Literature Evaluation

The quality of the literature was assessed using the risk of bias assessment tool provided by the Cochrane Collaboration. The following items were considered: (1) the generation of the random assignment plan, (2) whether the patients and doctors were blinded, (3) whether the outcome evaluation was blinded or not, (4) whether it was a hidden allocation scheme, (5) whether it was a selectively reported study result, (6) whether the result data were complete or not, and (7) whether there was other bias.

The quality of the included literature was evaluated on a scale of 1 to 7 using the modified Jadad scale from the aspects of random sequence generation, randomization concealment, blinding, and complete follow-up. Scores 1–3 were considered low quality and 4–7 high quality.

### 2.4. Data Extraction

According to the above principles, two researchers independently searched and screened the literature and evaluated the quality of the final included results using EndNote software. In case of any disagreement, the third researcher would join and make a decision.

### 2.5. Statistical Processing

Body weight and BMI were numerical variables. The difference between the variables before and after treatment was used to calculate the standard deviation of the variable difference with the help of the correlation coefficient (*R* = 0.5), and 95% confidence intervals (CI) of the median and percentile of the difference were estimated. Effectiveness was count data, for which the odds ratio (OR) was used for statistical analysis, and with 95% CI were calculated. The heterogeneity analysis was performed using the *I*^2^ value. If *I*^2^<50%, the heterogeneity was small and could be ignored, and the fixed effect model was used. If *I*^2^>50%, the heterogeneity was large, and the random effect model was used.

STATA15.0 software was used to draw the network evidence relationship diagram, forest plot, rank probability diagram, and funnel plot with corresponding statistics, and the consistency test was used to compare the ring consistency. In this study, the surface under the cumulative ranking curve (SUCRA) was used to calculate the cumulative ranking probability of each treatment regimen. A higher SUCRA value indicates more effective intervention.

## 3. Literature Search Results

### 3.1. Literature Search

**Figure 1:**
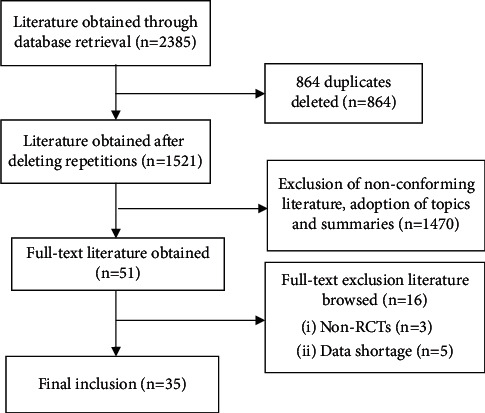
Flowchart of literature screening.

Using the above retrieval strategies, 2385 studies were retrieved from the databases, 864 duplicate studies were deleted, 1470 studies that obviously did not meet the inclusion criteria were excluded according to the title and abstract, and 51 were initially included. After intensive reading of the full text, 16 substandard studies were excluded, and finally 35 RCTs were included. The document screening process is shown in Figure 1.

### 3.2. Basic Information of Included Literature

The included 35 RCTs were all from China, with a total of eligible 3040 patients. Acupoint catgut embedding, acupuncture, electroacupuncture, TCM, acupoint catgut embedding + acupuncture, acupoint catgut embedding + exercise diet therapy, acupoint catgut embedding + TCM, exercise diet therapy, acupoint catgut embedding + moxibustion, and acupoint catgut embedding + cupping were used mainly for treatment. The basic characteristics of the included literature studies are shown in Table 1.

### 3.3. Literature Quality Research

In the included literature, there were 15 studies [12, 16, 18, 19, 21, 22, 24, 26, 28, 34, 36, 38, 39, 41, 42] using the random number table method, 16 studies [10, 11, 13–15, 17, 20, 23, 25, 27, 29–32, 37, 40] only presented random, 1 study [33] used the treatment method, 1 study [8] adopted the odd-even numbering method, 1 study [9] used the envelope assignment method, 1 study [35] used the odd and even admission numbers, 1 study [24] mentioned single-blindness, no study mentioned allocation concealment, and all study results data were complete. See Table 2 for details.

### 3.4. Traditional Meta-Analysis Results

Meta-analysis showed that the total effective rate of acupoint catgut embedding + TCM treatment, acupoint catgut embedding + moxibustion treatment, and acupoint catgut embedding + cupping treatment was higher than that of acupoint catgut embedding treatment alone with statistically significant differences. Acupoint catgut embedding and acupoint catgut embedding + acupuncture were superior to acupuncture treatment, and the differences were statistically significant. Acupoint catgut embedding + TCM treatment was more effective than TCM treatment with statistically significant differences. Acupoint catgut embedding was superior to electroacupuncture, and the difference was statistically significant; other direct comparisons showed no significant difference.

Meta-analysis results of weight loss showed that acupoint catgut embedding + TCM treatment, acupoint catgut embedding + moxibustion treatment, and acupoint catgut embedding + cupping treatment were superior to acupoint catgut embedding, and the difference was statistically significant. Acupoint catgut embedding was superior to acupuncture, and the difference was statistically significant. Acupoint catgut embedding + TCM treatment was superior to TCM treatment, and the difference was statistically significant. Other direct comparisons showed no statistically significant differences.

Meta-analysis results showed that BMI reduction by acupoint catgut embedding + TCM treatment, acupoint catgut embedding + moxibustion treatment, and acupoint catgut embedding + cupping treatment were more evident than that by acupoint catgut embedding treatment alone, acupoint embedding + TCM treatment was superior to TCM treatment, acupoint embedding treatment was superior to acupuncture, acupoint catgut embedding was superior to electroacupuncture, acupoint catgut embedding + exercise diet therapy was superior to exercise diet therapy, and there was no significant difference in other direct comparison results. The meta-analysis results are shown in Table 3.

### 3.5. Results of Network Meta-Analysis

#### 3.5.1. Evidence Network

The results of the total effective rate were as follows: the acupoint embedding was the center point, and the star-shaped structure of 10 intervention nodes formed 6 triangular closed loops, which were acupoint embedding-acupuncture-acupoint embedding + acupuncture, acupoint embedding-electrical acupuncture-exercise diet therapy, acupoint embedding-acupuncture-acupoint embedding + TCM, acupoint embedding-acupuncture-acupoint embedding + cupping, acupoint embedding-TCM-acupoint embedding + TCM, and acupoint embedding-acupoint embedding + exercise diet therapy-exercise diet therapy.

The reduction of body mass was as follows: the star-shaped structure of 9 intervention nodes centered on acupoint embedding, forming a total of 5 triangular closed loops, namely, acupoint embedding-acupuncture-acupoint embedding + TCM, acupoint embedding-acupuncture needling-acupoint catgut embedding + cupping, acupoint catgut embedding-electroacupuncture-exercise diet therapy, acupoint catgut embedding-TCM-acupoint catgut embedding + TCM, and acupoint catgut embedding-acupoint catgut embedding + exercise diet therapy-exercise diet therapy.

**Figure 2:**
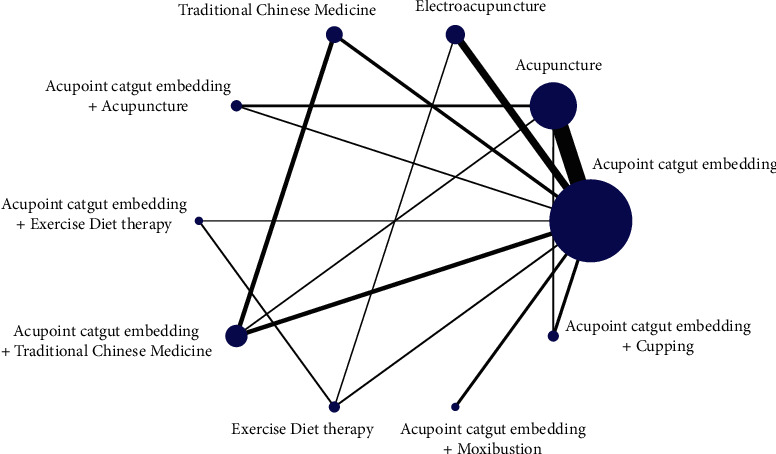
Evidence graph of total effective rate by network meta-analysis of different therapies and catgut embedding at acupoints in the treatment of simple obesity.

**Figure 3:**
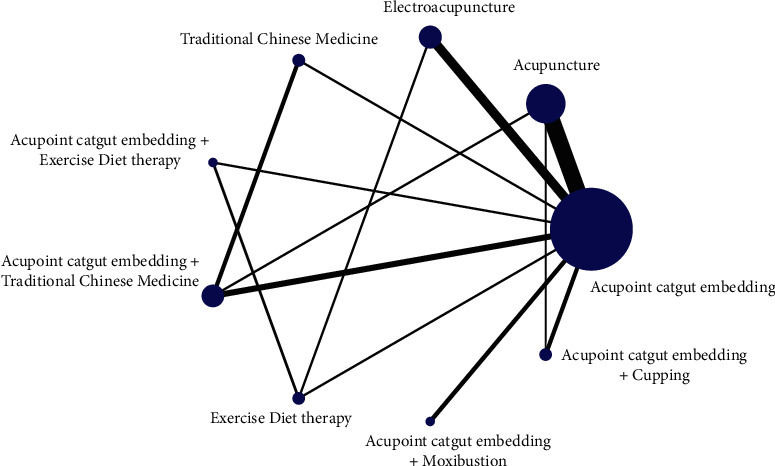
Evidence chart of weight loss by network meta-analysis of different therapies and catgut embedding at acupoints for simple obesity.

**Figure 4:**
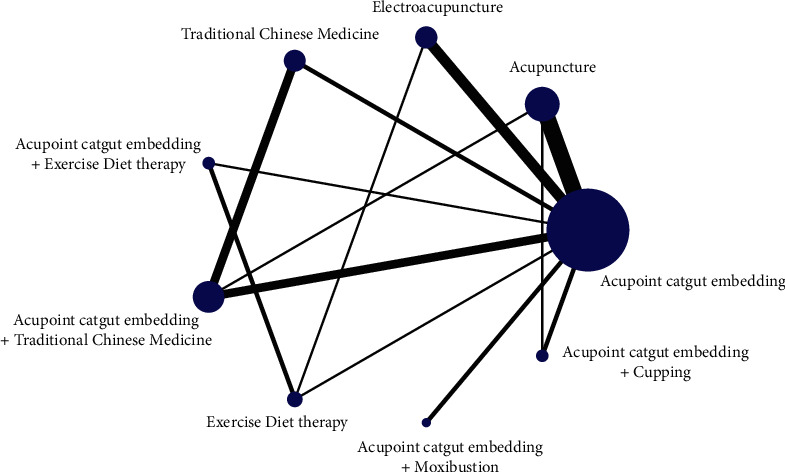
Evidence graph of BMI reduction by network meta-analysis of different therapies and catgut embedding at acupoints for simple obesity.

The reduction of BMI was as follows: the star-shaped structure with 9 intervention nodes centered on acupoint embedding, forming a total of 5 triangular closed loops, namely, acupoint embedding-acupuncture-acupoint embedding + TCM, acupoint embedding-acupuncture-catgut embedding + cupping, catgut embedding-electroacupuncture-sports diet therapy, acupoint catgut-TCM-acupoint catgut embedding + TCM, and acupoint catgut-acupoint catgut embedding + exercise diet therapy-exercise diet therapy. The network evidence graph results are shown in Figures 2–4.

#### 3.5.2. Consistency Test

The variable of the total effective rate of treatment contained 6 closed loops, the lower limit of the 95% CI of the 5 loop inconsistency factors (IFs) after the consistency test was 0, there was no obvious inconsistency, and the 95% lower limit of 1 closed loop IF was 0.16, failing to reach 0. Inconsistency was statistically significant, indicating inconsistency.

The decrease in body mass involved 5 closed loops, the lower limit of the 95% CI of the discordance factor IF contained 0, and there was no significant discordance.

**Figure 5:**
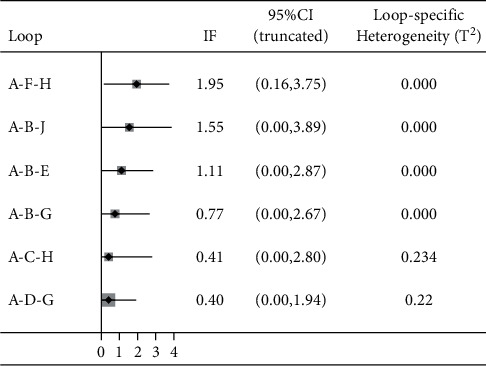
Inconsistency test results of total effective rate. A: acupoint catgut embedding; B: acupuncture; C: electroacupuncture; D: TCM; E: acupoint catgut embedding + acupuncture; F: acupoint catgut embedding + exercise diet therapy; G: acupoint catgut embedding + TCM; H: exercise diet therapy; J: acupoint catgut embedding + cupping.

**Figure 6:**
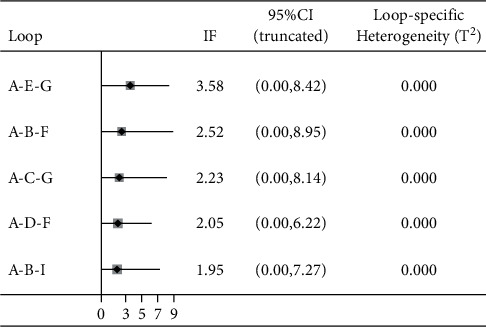
Inconsistency test results of body weight reduction. A: acupoint catgut embedding; B: acupuncture; C: electroacupuncture; D: TCM; E: acupoint catgut embedding + acupuncture; F: acupoint catgut embedding + exercise diet therapy; G: acupoint catgut embedding + TCM; I: acupoint catgut embedding + moxibustion.

**Figure 7:**
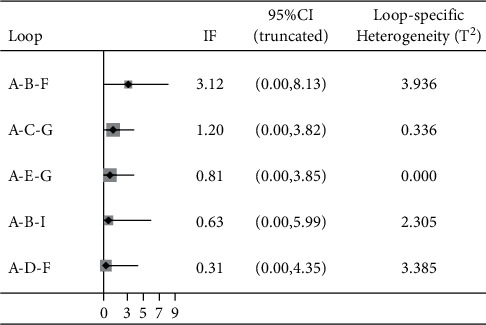
Results of inconsistency test of BMI reduction. A: acupoint catgut embedding; B: acupuncture; C: electroacupuncture; D: TCM; E: acupoint catgut embedding + acupuncture; F: acupoint catgut embedding + exercise diet therapy; G: acupoint catgut embedding + TCM; I: acupoint catgut embedding + moxibustion.

BMI reduction involved 5 closed loops, the lower limit of the 95% CI of the discordance factor IF contained 0, and there was no significant discordance. The results of the consistency check are shown in Figures 5–7.

#### 3.5.3. Results of Network Meta-Analysis

**Figure 8:**
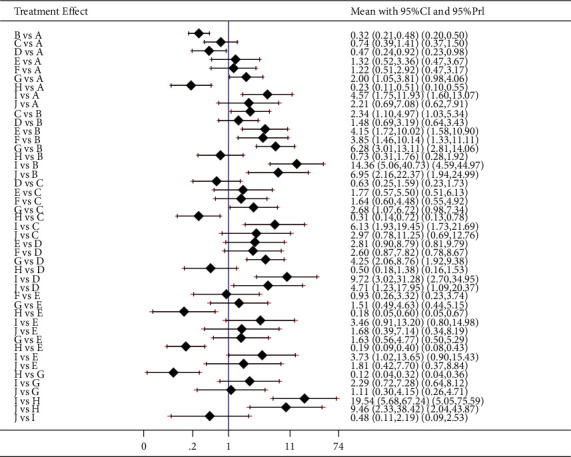
The total effective rate in treatment of simple obesity with different methods and catgut embedding at acupoints in meta-analysis forest plot. A: acupoint catgut embedding; B: acupuncture, C: electroacupuncture; D: TCM; E: acupoint catgut embedding + acupuncture; F: acupoint catgut embedding + exercise diet therapy; G: acupoint catgut embedding + TCM; H: exercise diet therapy; I: acupoint catgut embedding + moxibustion; J: acupoint catgut embedding + cupping.

The results of the total effective rate were as follows: the healing of acupoint catgut embedding was better than that of acupuncture, electroacupuncture, and exercise diet therapy (*P* < 0.05). The total effective rate of acupoint catgut embedding + moxibustion was higher than that of acupoint catgut embedding (*P* < 0.05); electroacupuncture, catgut embedding + acupuncture, acupoint catgut + exercise diet therapy, acupoint catgut + TCM, acupoint catgut + moxibustion, and acupoint catgut + cupping were superior to acupuncture (*P* < 0.05); the total effective rate of acupoint catgut embedding + moxibustion was higher than that of electroacupuncture (*P* < 0.05); the total effective rate of acupoint catgut embedding + Chinese medicine, acupoint catgut embedding + moxibustion, and acupoint catgut embedding + cupping was higher than that of TCM treatment (*P* < 0.05); and electroacupuncture, acupoint catgut embedding, acupoint catgut embedding + acupuncture, acupoint catgut embedding + sports diet therapy, acupoint catgut embedding + TCM, acupoint catgut embedding + moxibustion, and acupoint catgut embedding + cupping were superior to sports diet therapy (*P* < 0.05). The results are shown in Figure 8.

**Figure 9:**
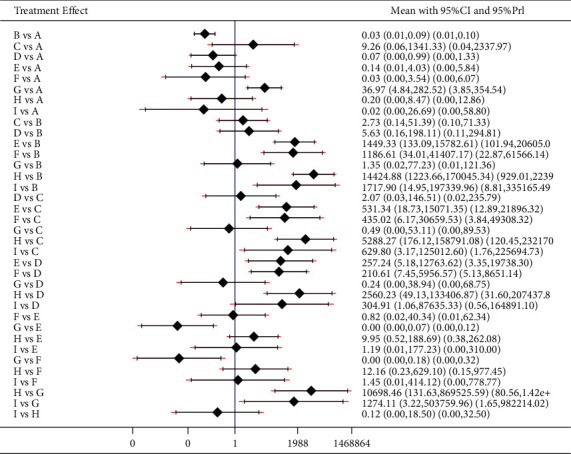
Weight loss in treatment of simple obesity with different methods and catgut embedding at acupoints in meta-analysis forest plot. A: acupoint catgut embedding; B: acupuncture; C: electroacupuncture; D: TCM; E: acupoint catgut embedding + exercise diet therapy; F: acupoint catgut embedding + TCM; G: exercise diet therapy; H: acupoint catgut embedding + moxibustion; I: acupoint catgut embedding + cupping.

The results of weight loss were as follows: acupuncture treatment was superior to acupoint catgut embedding therapy (*P* < 0.05); acupoint catgut embedding + exercise diet therapy, acupoint catgut embedding + TCM, acupoint catgut embedding + moxibustion, and acupoint catgut embedding + cupping were superior to acupuncture and electroacupuncture treatment (*P* < 0.05); acupoint catgut embedding + exercise diet therapy, acupoint catgut embedding + TCM, and acupoint catgut embedding + moxibustion were superior to TCM treatment (*P* < 0.05); and acupoint catgut embedding, acupoint catgut embedding + acupuncture, acupoint catgut embedding + exercise diet therapy, catgut embedding + TCM, acupoint catgut embedding + moxibustion, and acupoint catgut embedding + cupping were superior to exercise diet therapy (*P* < 0.05). The results are shown in Figure 9.

**Figure 10:**
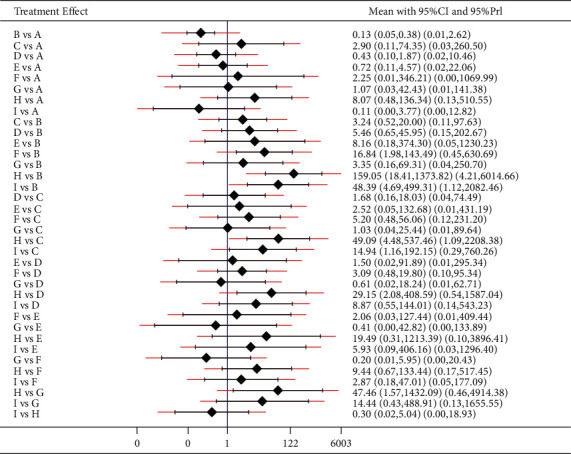
BMI reduction in treatment of simple obesity with different methods and catgut embedding at acupoints in meta-analysis forest plot. A: acupoint catgut embedding; B: acupuncture; C: electroacupuncture; D: TCM; E: acupoint catgut embedding + exercise diet therapy; F: acupoint catgut embedding + TCM; G: exercise diet therapy; H: acupoint catgut embedding + moxibustion; I: acupoint catgut embedding + cupping.

The results of BMI reduction were as follows: acupoint catgut embedding + moxibustion and acupoint catgut embedding + cupping were superior to acupuncture treatment (*P* < 0.05); and acupoint catgut embedding + moxibustion was superior to electroacupuncture treatment (*P* < 0.05). The results are shown in Figure 10.

#### 3.5.4. Sorting of Mesh Meta-Analysis Results

Three different outcome indicators were ranked, and there were some differences in the ranking results. Lower average rank indicates better outcome indicators. Finally, it was revealed the intervention measures of acupoint catgut embedding combined with moxibustion showed a better effect in the treatment of simple obesity. The ranking of network meta-analysis results is shown in Table 4.

### 3.6. Publication Bias

**Figure 11:**
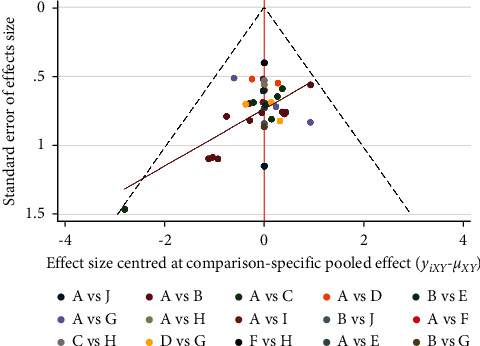
Comparison of total effective rate of treatment of simple obesity-corrected funnel plot. A: acupoint catgut embedding; B: acupuncture; C: electroacupuncture; D: TCM; E: acupoint catgut embedding + acupuncture; F: acupoint catgut embedding + exercise diet therapy; G: acupoint catgut embedding + TCM; H: exercise diet therapy; I: acupoint catgut embedding + moxibustion; J: acupoint catgut embedding + cupping.

The funnel plot was drawn according to the total effective rate, and the scatter points were mostly located in the upper half, symmetrically distributed on both sides of the red indicator line, indicating small publication bias. However, there was a scatter at the bottom of the funnel plot, indicating a small sample effect, as shown in Figure 11.

## 4. Discussion

Simple obesity is defined as malnutrition without obvious causes. When the accumulation of body fat exceeds the consumption level of the body, the patient's weight exceeds the standard weight due to excessive body fat [43]. Nowadays, many factors are considered to be the etiology of obesity, such as neuroregulation, free radicals, and heredity [44, 45]. In traditional Chinese medicine, it is believed that dysfunction of the spleen and stomach is the root cause of obesity. Increasing intake of sweet and greasy food and declining function of the spleen and stomach leads to accumulation of fat in the body. Obesity affects the quality of life of patients and damages their physical and mental health. In clinical reports, acupoint catgut embedding is a safe and effective intervention for obesity.

Adipocytes, adipose tissue, endocrine regulation, and inflammatory factors are the focus of study on the mechanism of action of acupoint catgut embedding in the treatment of simple obesity [46]. In the process of acupoint catgut embedding, needle insertion can cause tissue damage, fat cell death, or a small range of fat liquefaction and, to a certain extent, can reduce the number of cells in adipose tissue [47]. Under an optical microscope, less adipocytes, less lipid droplets in the cytoplasm, uniform cells, and more compact adipocytes were observed in obese mice [48]. Leptin (LP), as a product of adipocyte secretion, is a peptide hormone that acts on multiple tissues and organs through its receptor and has multiple effects on regulation of the body. Yan Runhu [49] treated rats fed with high-fat diet for 12 weeks with catgut implantation at acupoints. The results showed that catgut implantation at acupoints could upregulate the expression of OB-Rb mRNA in the hypothalamus of obese rats and reduce the expression of SOCS-3 mRNA in hypothalamus cells and the content of SOCS-3 in peripheral blood serum, which suggested that catgut implantation at acupoints promoted signal transduction after LP and fasting insulin receptors, improved leptin resistance (LR) and insulin resistance (IR), and promoted LP and fasting insulin to exert biological effects. Therefore, it can be used as an important mechanism of acupoint catgut embedding for weight loss. Deng Min [50] observed the effect of catgut implantation at acupoints on inflammatory factors in mice and found that catgut implantation at acupoints could inhibit the expression of interleukin-mRNA, tumor necrosis factor-*α*-mRNA, and monocyte chemoattractant protein-1 mRNA in adipose tissue and could reduce the occurrence and development of inflammatory reactions. Therefore, it is further predicted that the possible mechanism of weight loss by catgut implantation at acupoints is to increase the expression of inflammatory factors in adipose tissue. The meta-analysis of acupoint catgut embedding and related therapies for obesity revealed that acupoint catgut embedding and other therapies showed a high healing rate in the treatment of simple obesity and could reduce the BMI of the patients [6]. Acupoint catgut embedding has become one of the effective measures for the treatment of obesity and has shown good clinical results with a variety of combined therapies, which are widely used [51].

To investigate the curative effect of catgut implantation at each acupoint and related therapies, a network meta-analysis was conducted. A total of 35 studies were included in this study, including 3040 patients. There was a significant difference between the two groups before and after treatment. In network meta-analysis, the effect of various treatment methods was compared. Compared with traditional meta-analysis, it contains more original data. The statistical accuracy of different groups is not enough, but it has no impact on the final results of network meta-analysis [47]. This study ranked the improvement of treatment effect, body mass, and BMI of patients with simple obesity by comparing the treatments including acupoint catgut embedding, acupuncture, TCM, electroacupuncture, and exercise diet therapy alone, as well as the combination of acupoint catgut embedding with different therapies. The results of network meta-analysis showed that, in terms of total effective rate, the top three were acupoint catgut embedding + moxibustion, acupoint catgut embedding + TCM, and acupoint catgut embedding + cupping; in terms of reduced body mass of patients, the top three were acupoint catgut embedding + moxibustion, acupoint catgut embedding + exercise and diet therapy, and acupoint catgut embedding + cupping; and in terms of reduced BMI of patients, the top three were acupoint catgut embedding + moxibustion, acupoint catgut embedding + TCM, and acupoint catgut embedding + cupping. Based on the results of network meta-analysis of the three indexes, acupoint catgut embedding and its combination with other therapies were the best treatments for simple obesity. There was no obvious asymmetry in the comparison correction funnel chart, indicating no publication bias, but there was a scatter at the bottom, indicating the influence of small samples. The inconsistency test indicated good consistency of each closed loop. However, the use of different acupoints, treatment courses, and drugs in the studies leads to clinical heterogeneity, which needs more high-quality RCT studies to verify.

There are some limitations in this study. Firstly, there are adverse reactions in the included literature, so it may cause bias. Secondly, some studies do not mention the random sequence method and do not blind the subjects and doctors, which may have an impact on the efficacy results. Thirdly, most of the observation indexes were body mass, effective rate, BMI, WC, HC, waist-hip ratio, and so on, lacking objective laboratory indexes. Finally, the short-term effect is good, but there are few follow-up records. The long-term effect needs to be further discussed. This systematic review aims to update and improve.

## 5. Conclusion

Acupoint catgut embedding and its combination with different therapies significantly increase the effective rate in treatment of simple obesity, resulting in improved body mass and BMI of the patients. The use of acupoint catgut embedding therapy is a better choice and provides a more reliable clinical reference. In clinical treatment, acupoint catgut embedding can be selected based on the conclusion of this study and considering syndrome differentiation. However, the conclusion is affected by the quality of the included studies, and this study needs more high-quality, large-sample RCT studies to verify.

## Figures and Tables

**Table 1:** Basic characteristics of included studies.

Author and year	Trial 1			Trial 2			Trial 3			Period of treatment (week)	Evaluation standard course of disease
	Interventions	Number (male/female)	Age (years)	Interventions	Number (male/female)	Age (years)	Interventions	Number (male/female)	Age (years)		
Luo Liangqi 2016 [8]	Acupoint catgut embedding	30 (11/19)	32.8 ± 3.6	Acupuncture	30 (10/20)	31.6 ± 4.3				4/4	(1)
Zhou Wei 2020 [9]	Acupoint catgut embedding	45	21–45	Electroacupuncture	45	21–45	Exercise diet therapy	45	21–45	8/8/8	(1) (2) (3) (4) (5)
Li Miaomiao 2017 [10]	Acupoint catgut embedding	30 (4/26)	18–58	Electroacupuncture	30 (4/26)	25–53				4/4	(1) (2) (3)
Zheng Xi 2020 [11]	Acupoint catgut embedding + moxibustion	48 (24/24)	41.97 ± 15.22	Acupoint catgut embedding	48 (25/23)	42.15 ± 15.69				8/8	(1) (2) (3) (6) (7) (8)
Duan Xiaorong 2017 [12]	Acupoint catgut embedding + cupping	50 (11/39)	35.48 ± 8.269	Acupuncture	50 (10/40)	35.14 ± 7.743				12/12	(1) (2) (3)
Wang Zheng 2020 [13]	Acupoint catgut embedding	56 (32/24)	43.3 ± 2.6	Acupuncture	56 (30/26)	43.5 ± 2.7				4/4	(1) (2) (3) (9)
Zhou Lijie 2017 [14]	Acupoint catgut embedding	33 (4/29)		Acupuncture	33 (2/31)					4/4	(1) (2) (3) (9)
Wu Xiaomei 2015 [15]	Acupoint catgut embedding	32 (7/25)	33 ± 11	Acupuncture	30 (6/24)	35 ± 10				4/4	(1) (2) (3) (5)
Huang Qiong 2020 [16]	Acupoint catgut embedding	39 (21/18)	38.27 ± 2.52	Acupuncture	39 (20/19)	37.34 ± 2.57				12/12	(2) (3) (7) (8)
Lin Guanghua 2015 [17]	Acupoint catgut embedding + cupping	30 (4/26)	32.56 ± 16.62	Acupoint catgut embedding	30 (3/27)	31.98 ± 17.05				8/8	(1) (2) (3)
Deng Ru 2021 [18]	Acupoint catgut embedding	30 (15/15)	58.5 ± 2.4	Acupuncture	30 (14/16)	47.7 ± 3.6				8/8	(1) (2) (3) (5)
Huang Wei 2015 [19]	Acupoint catgut embedding + exercise diet therapy	80 (0/80)	20–45	Acupoint catgut embedding	80 (0/80)	20–45				12/12	(1) (2) (3) (4)
Lin Chenjuan 2020 [20]	Acupoint catgut embedding + TCM	30 (17/13)	33.81 ± 6.32	Acupoint catgut embedding	30 (14/16)	33.12 ± 6.45	TCM	30 (16/14)	33.49 ± 6.70	12/12/12	(1) (2) (3) (5) (7) (8) (10) (11) (13)
Wen Qingfen 2021 [21]	Acupoint catgut embedding + TCM	41 (17/24)	33.48 ± 10.39	Acupoint catgut embedding	30 (18/23)	33.56 ± 10.52				8/8	(2) (3) (5) (6) (12) (14) (15)
Su Junxian 2017 [22]	Acupoint catgut embedding + TCM	38 (11/27)	38.05 ± 5.91	Acupoint catgut embedding	39 (14/15)	38.70 ± 6.16	TCM	38 (12/16)	38.45 ± 6.19	4/4/4	(1) (3) (5)
Wang Rui 2017 [23]	Acupoint catgut embedding + exercise diet therapy	30 (5/25)	32.45 ± 10.40	Exercise diet therapy	30 (7/23)	29.62 ± 7.25				8/12	(3) (6)
Wang Lingshu 2019 [24]	Acupoint catgut embedding + TCM	60 (28/32)	34.1 ± 7.42	Acupoint catgut embedding	60 (29/31)	34.0 ± 7.40				6/6	(1) (2) (3) (5) (6)
Chen Rongzhong 2016 [25]	Acupoint catgut embedding	47	42.8 ± 2.9	Acupuncture	47	42.8 ± 2.9				8/8	(1) (2) (5) (12) (16)
Zhou Hualing 2018 [26]	Acupoint catgut embedding + TCM	28 (4/24)	30.67 ± 2.48	Acupuncture	28 (3/25)	31.25 ± 2.07				16/4	(1) (2) (3) (5) (12)
Zheng Xiao 2015 [27]	Acupoint catgut embedding	40 (4/36)	15–56	Electroacupuncture	40 (3/37)	14–60				12/12	(1) (3) (5)
Guo Wenjiang 2014 [28]	Acupoint catgut embedding	36 (14/22)	33.6 ± 1.5	Acupuncture	35 (11/24)	34.2 ± 1.5				6/6	(1)
Zhang Hong 2017 [29]	Acupoint catgut embedding	40 (22/18)	61.35 ± 3.11	Acupuncture	40 (23/17)	61.26 ± 3.07				12/12	(1) (2) (3) (5) (7) (8) (10) (11) (12)
Zhao Huayi 2015 [30]	Acupoint catgut embedding	50 (2/48)	25–60	Electroacupuncture	50 (2/48)	23–60				4/6	(1) (2) (3) (5) (12)
Yao Rujie 2014 [31]	Acupoint catgut embedding	25 (10/15)	38.3 ± 9.83	Acupuncture	25 (9/16)	37.78 ± 9.27				12/12	(1) (4) (5) (6) (12) (17)
Chen Zeli 2013 [32]	Acupoint catgut embedding	40 (5/35)	39.65 ± 4.82	Acupuncture	40 (6/34)	38.95 ± 4.54				6/6	(1) (2) (3)
Huang Weixuan 2019 [33]	Acupoint catgut embedding + exercise diet therapy	100 (29/71)	48.9 ± 12.1	Exercise Diet therapy	100 (26/74)	49.3 ± 11.8				12/12	(1) (2) (3) (5) (7) (8) (10)
Yan Bing 2021 [34]	Acupoint catgut embedding	29 (11/18)	33.5 ± 9.4	Acupuncture	30 (10/20)	33.7 ± 10.2				8/8	(3) (5) (7) (8) (10) (11) (12) (14) (18)
Li Lujuan 2016 [35]	Acupoint catgut embedding	50 (20/30)	35.2 ± 12.6	Electroacupuncture	50 (19/31)	36.7 ± 11.8				8/8	(1) (2) (3)
Wang Quan 2018 [36]	Acupoint catgut embedding + TCM	50 (26/24)	31.32 ± 2.72	TCM	50 (26/24)	31.31 ± 2.72				12/12	(2) (3) (5) (7) (8) (10)
Zhao Binbin 2015 [37]	Acupoint catgut embedding + cupping	30 (4/26)	32.56 ± 16.62	Acupoint catgut embedding	30 (3/27)	31.98 ± 17.05				8/8	(1) (2) (3) (5)
Liang Bingjun 2019 [38]	Acupoint catgut embedding + TCM	40 (24/16)	41.23 ± 7.41	TCM	40 (25/15)	41.25 ± 7.59				4/4	(1) (2) (3)
Lv Mingfang 2020 [39]	Acupoint catgut embedding + moxibustion	40 (25/15)	30.7 ± 4.1	Acupoint catgut embedding	40 (23/17)	31.5 ± 3.7				8/8	(1) (2) (3) (5) (6) (12) (17) (19)
Chen Yuanyuan 2015 [40]	Acupoint catgut embedding + acupuncture	40	18–46	Acupoint catgut embedding	40	18–46	Acupuncture	40	18–46	4/4/4	(1)
Hou Sujuan 2016 [41]	Acupoint catgut embedding + acupuncture	68 (44/24)	27.1 ± 1.5	Acupuncture	68 (45/13)	26.8 ± 1.2				5/5	(1) (4) (6) (8)
Tian Feng 2014 [42]	Acupoint catgut embedding	22	26–49	Acupuncture	22	26–49				8/8	(1)

*Note*. TCM: traditional Chinese medicine; (1): effective rate; (2): body weight (kg); (3): BMI (kg/m2); (4): body fat percentage (%); (5): waistline (cm); (6): WHR (waist-hip ratio); (7): TC; (8): TG; (9): appetite score; (10): LDL-C; (11): HDL-C; (12): hip circumference; (13): adverse reaction; (14): fat thickness; (15): Chinese medicine syndrome scores; (16): chest circumference; (17): plumpness; (18): IWQOL-Lite score; (19): body fat percentage.

**Table 2:** Literature quality research.

Study	Stochastic method	Randomized hiding	Blinding	Results data integrity	Jadad score
Luo Liangqi 2016 [8]	Parity number	Unclear	Unclear	Integrity	5
Zhou Wei 2020 [9]	Envelope drawing method	Unclear	Unclear	Integrity	5
Li Miaomiao2017 [10]	Random	Unclear	Unclear	Integrity	4
Zheng Xi 2020 [11]	Random	Unclear	Unclear	Integrity	4
Duan Xiaorong 2017 [12]	Random number list	Unclear	Unclear	Integrity	5
Wang Zheng 2020 [13]	Random	Unclear	Unclear	Integrity	4
Zhou Lijie 2017 [14]	Random	Unclear	Unclear	Integrity	4
Wu Xiaomei 2015 [15]	Random	Unclear	Unclear	Integrity	4
Huang Qiong 2020 [16]	Random number list	Unclear	Unclear	Integrity	5
Lin Guanghua 2015 [17]	Random	Unclear	Unclear	Integrity	4
Deng Ru 2021 [18]	Random number list	Unclear	Unclear	Integrity	4
Huang Wei 2015 [19]	Random number list	Unclear	Unclear	Integrity	5
Lin Chenjuan 2020 [20]	Random	Unclear	Unclear	Integrity	4
Wen Qingfen 2021 [21]	Random number list	Unclear	Unclear	Integrity	5
Su Junxian 2017 [22]	Random number list	Unclear	Unclear	Integrity	5
Wang Rui 2017 [23]	Random	Unclear	Unclear	Integrity	4
Wang Lingshu 2019 [24]	Random number list	Unclear	Unclear	Integrity	6
Chen Rongzhong 2016 [25]	Random	Unclear	Unclear	Integrity	4
Zhou Hualing 2018 [26]	Random number list	Unclear	Unclear	Integrity	5
Zheng Xiao 2015 [27]	Random	Unclear	Unclear	Integrity	4
Guo Wenjiang 2014 [28]	Random number list	Unclear	Unclear	Integrity	5
Zhang Hong 2017 [29]	Random	Unclear	Unclear	Integrity	4
Zhao Huayi 2015 [30]	Random	Unclear	Unclear	Integrity	4
Yao Rujie 2014 [31]	Random	Unclear	Unclear	Integrity	4
Chen Zeli 2013 [32]	Random	Unclear	Unclear	Integrity	4
Huang Weixuan 2019 [33]	Therapies	Unclear	Unclear	Integrity	3
Yan Bing 2021 [34]	Random number list	Unclear	Unclear	Integrity	5
Li Lujuan 2016 [35]	Single and double numbers	Unclear	Unclear	Integrity	5
Wang Quan 2018 [36]	Random number list	Unclear	Unclear	Integrity	5
Zhao Binbin 2015 [37]	Random	Unclear	Unclear	Integrity	4
Liang Bingjun 2019 [38]	Random number list	Unclear	Unclear	Integrity	5
Lv Mingfang 2020 [39]	Random number list	Unclear	Unclear	Integrity	5
Chen Yuanyuan 2015 [40]	Random	Unclear	Unclear	Integrity	4
Hou Sujuan 2016 [41]	Random number list	Unclear	Unclear	Integrity	5
Tian Feng 2014 [42]	Random number list	Unclear	Unclear	Integrity	5

**Table 3:** Traditional meta-analysis results.

Interventions	Number of studies included	OR/MD（95% CI）	*P*	*χ* ^2^	*P*	*I* ^2^
*Total effective rate*						
Acupoint catgut embedding vs. acupuncture	12	3.77 (2.49, 5.71)	≤0.001	7.56	0.75	0%
Acupoint catgut embedding vs. electroacupuncture	5	1.99 (1.10, 3.61)	0.02	4.82	0.31	17%
Acupoint catgut embedding + TCM vs. acupoint catgut embedding	3	2.32 (1.14, 4.72)	0.02	2.70	0.26	26%
Acupoint catgut embedding + TCM vs. TCM	3	5.99 (2.63, 13.66)	≤0.001	0.46	0.80	0%
Acupoint catgut embedding + moxibustion vs. acupoint catgut embedding	2	4.57 (1.75, 11.92)	0.002	0.00	0.96	0%
Acupoint catgut embedding + cupping vs. acupoint catgut embedding	2	4.46 (0.91, 21.97)	0.07	0.00	1.00	0%
Acupoint catgut embedding + acupuncture vs. acupuncture	2	3.49 (1.42, 8.61)	0.007	0.00	0.96	0%
Acupoint catgut embedding + exercise diet therapy vs. exercise diet therapy	1	3.33 (1.51, 7.32)	0.003			
Acupoint catgut embedding + exercise diet therapy vs. acupoint catgut embedding	1	3.35 (1.03, 10.89)	0.04			
Acupoint catgut embedding + TCM vs. acupuncture	1	3.27 (0.63, 17.07)	0.16			
Acupoint catgut embedding + cupping vs. acupuncture	1	3.55 (0.65, 19.37)	0.14			

*Body weight*						
Acupoint catgut embedding vs. acupuncture	9	−3.86 (−5.56, −2.61)	≤0.001	19.16	0.01	58%
Acupoint catgut embedding vs. electroacupuncture	4	−0.34 (−3.11, 2.43)	0.81	0.99	0.80	0%
Acupoint catgut embedding + TCM vs. acupoint catgut embedding	3	−2.04 (−3.24, −0.84)	≤0.001	1.24	0.54	0%
Acupoint catgut embedding + TCM vs. TCM	3	−5.61 (−7.21, −4.01)	≤0.001	0.35	0.84	0%
Acupoint catgut embedding + moxibustion vs. acupoint catgut embedding	2	−4.96 (−6.26, −3.67)	≤0.001	1.24	0.27	19%
Acupoint catgut embedding + cupping vs. acupoint catgut embedding	2	−4.14 (−8.18, −0.10)	0.04	0.00	1.00	0%
Acupoint catgut embedding + exercise diet therapy vs. exercise diet therapy	1	−4.20 (−6.45, −1.95)	≤0.001			
Acupoint catgut embedding + exercise diet therapy vs. acupoint catgut embedding	1	−2.78 (−4.47, −1.09)	0.001			
Acupoint catgut embedding + TCM vs. acupuncture	1	−5.44 (−7.85, −3.03)	≤0.001			
Acupoint catgut embedding + cupping vs. acupuncture	1	−2.85 (−9.33, 3.63)	0.39			

*BMI*						
Acupoint catgut embedding vs. acupuncture	8	−1.84 (−2.23, −1.44)	≤0.001	134.27	0.000	95%
Acupoint catgut embedding vs. electroacupuncture	5	−0.47 (−1.11, 0.17)	0.15	7.56	0.11	47%
Acupoint catgut embedding + TCM vs. acupoint catgut embedding	4	−1.30 (−2.33, −0.27)	0.01	50.67	0.000	94%
Acupoint catgut embedding + TCM vs. TCM	4	−1.56 (−2.30, −0.82)	≤0.001	18.60	0.000	84%
Acupoint catgut embedding + moxibustion vs. acupoint catgut embedding	2	−2.69 (−3.22, −2.16)	≤0.001	0.04	0.84	0%
Acupoint catgut embedding + cupping vs. acupoint catgut embedding	2	−1.92 (−2.90, −0.94)	≤0.001	0.00	1.00	0%
Acupoint catgut embedding + exercise diet therapy vs. exercise diet therapy	2	−1.66 (−2.16, −1.15)	≤0.001	0.36	0.55	0%
Acupoint catgut embedding + exercise diet therapy vs. acupoint catgut embedding	1	−2.18 (−5.25, 0.89)	0.16			
Acupoint catgut embedding + TCM vs. acupuncture	1	−4.82 (−5.75, −3.89)	≤0.001			
Acupoint catgut embedding + cupping vs. acupuncture	1	−0.98 (−1.87, −0.09)	0.03			

**Table 4:** The sorting table of network meta-analysis results of different treatment methods and catgut embedding at acupoints for simple obesity.

Interventions	Total efficacy ranking		Weight loss ranking		BMI reduction ranking	
	SUCRA	Rank	SUCRA	Rank	SUCRA	Rank
Acupoint catgut embedding	50.7	6	48.4	5	47.9	4
Acupuncture	10.3	9	8.4	9	7.4	8
Electroacupuncture	37.7	7	32.0	6	31.3	7
TCM	22.2	8	10.8	8	36.3	6
Acupoint catgut embedding + acupuncture	61.6	4				
Acupoint catgut embedding + exercise diet therapy	59.9	5	77.1	2	53.9	5
Acupoint catgut embedding + TCM	79.3	2	72.4	4	73.5	3
Exercise diet therapy	4.1	10	26.4	7	17.2	9
Acupoint catgut embedding + moxibustion	96.5	1	99.5	1	95.5	1
Acupoint catgut embedding + cupping	77.8	3	75.0	3	87.1	2

## Data Availability

The data used to support the findings of this study are available from the corresponding author upon request.
